# Morphological variations and morphometry details of the foramen ovale in the Saudi population: a retrospective radiological study

**DOI:** 10.25122/jml-2022-0265

**Published:** 2023-03

**Authors:** Mohammed Alaftan, Sajedah Alkhater, Fatima Alhaddad, Aqeelah Alfaraj, Noor Alrashed, Sanket Hiware, Ibrahim Alghnimi, Roaa Algowiez, Essam Ismail

**Affiliations:** 1 Radiology Department, Imam Abdulrahman Bin Faisal University, College of Medicine, Dammam, Saudi Arabia; 2 Imam Abdulrahman Bin Faisal University, College of Medicine, Dammam, Saudi Arabia; 3 Anatomy Department, Imam Abdulrahman Bin Faisal University, College of Medicine, Dammam, Saudi Arabia

**Keywords:** foramen ovale, morphological variations, morphometric dimensions, skull

## Abstract

The foramen ovale is one of the essential foramina in the middle cranial fossa, more precisely, in the superior surface of the greater wing of the sphenoid bone. It has essential surgical and diagnostic significance since it serves as a surgical landmark, and crucial neurovascular vessels such as the mandibular nerve and accessory meningeal artery pass through it. Therefore, understanding the morphological and morphometric variations of the foramen ovale is essential for accurately identifying, diagnosing, and managing related pathologies. The study aimed to evaluate the morphological variations and morphometric details of the foramen ovale in the Saudi population. A radiological study was conducted to observe the measurements and the shapes of the foramen ovale in the skull with its anatomical variants. Retrospective data was collected from the Department of Radiology, King Fahad University Hospital, Saudi Arabia. The sample consisted of 100 human skulls from computed tomography scans, including 50 males and 50 females. The values for the mean length, width, and distance from the midline on the right side were 6.462 mm ± 1.681 mm, 4.897 ± 1.0631 mm, and 2.4565 ± 0.51275 mm, and 6.451 ± 1.6691 mm, 4.812 ± 1.0848 mm and 2.4290 ± 0.60039 mm for the left side, respectively. The foramen shape was oval in the majority (47%), followed by round shape (31%) with no bony outgrowths such as spur in the studied foramina. Furthermore, the morphometric variation between males and females was statistically insignificant (p-value>0.05). The observed variation of foramen ovale in this study has significant anatomical and clinical applications for various diagnostic and surgical procedures.

## INTRODUCTION

From an anatomical perspective, the base of the human skull is divided into three main cranial fossae: anterior, posterior, and middle. Each fossa carries several important foramina that facilitate passing significant neurovascular supplies. The middle cranial fossa contains a foramen that is called “foramen ovale (FO),” located in the superior surface of the greater wing of the sphenoid bone and opening into the infratemporal fossa [1]. It acts as a transitional area between the extracranial and intracranial structures [2,3]. FO transmits the following nerves: the mandibular division of the trigeminal nerve and the lesser petrosal nerve [1]. In addition, the vascular structures are the accessory meningeal artery and the emissary vein [2].

Moreover, since all foramina in the skull contain venous plexuses, the cavernous sinus is connected with the pterygoid plexus by the venous plexus of FO [4]. However, these venous structures might be isolated from other passing structures in the FO by an anteromedial bony outgrowth called "spur". These spurs can compress the mandibular nerve and affect the supplied muscles [2].

The shape of the foramen ovale (FO) in the human skull can vary and may include bony projections, which can impact the structures passing through it [5]. FO can take on different shapes such as oval, round, almond, and tear-drop, and these variations are significant in surgical and diagnostic settings since it serves as crucial landmark during surgery.[2,5]. For instance, a careful examination of these foramina guides the diagnosis of the nasopharynx and middle cranial fossa lesions, such as fifth cranial nerve neuroma, that can lead to FO enlargement. In addition, it helps during the administration of anesthesia to the mandibular nerve, and it plays a significant role for neurosurgeons in some diagnostic and therapeutic procedures, such as microvascular decompression, a percutaneous biopsy of cavernous sinus tumors, percutaneous trigeminal rhizotomy, and seizure electroencephalographic analysis [2,6,7].

A comprehensive understanding of the characteristics and location of the FO, including its shape and potential variations, as well as the structures that pass through it, is crucial in preventing potential complications during intervention procedures. For instance, understanding the anatomy of FO can help prevent trigeminal nerve injury during neurosurgical procedures [6]. Therefore, it is of scientific importance to study the morphological and morphometric diversities of FO to identify, diagnose, and manage related pathologies. As there is a lack of research on the morphological variation of the FO, we conducted this study to provide preliminary data on this topic.

This study aimed to analyze the morphological variations and morphometric details of the foramen ovale in the Saudi population. The expected variations in morphology and the presence of bony outgrowths between the right and left sides, as well as between males and females, may be associated with the compression of neurovascular structures passing through the foramen. Therefore, the findings of this study could serve as a valuable reference for clinicians in understanding the clinical implications of the foramen ovale.

## MATERIAL AND METHODS

### Study type and participants

The retrospective cross-sectional study was conducted between March 2021 and March 2022 at the Department of Radiology, King Fahad Hospital of the University (KFHU), Al Khobar, Eastern Province, Saudi Arabia. The radiological data were obtained randomly from the picture archiving and communication system (PACS), where we retrieved CT images of Saudi patients performed for medical or surgical indications. Subjects with foramen ovale pathologies were excluded, along with any motion artifacts.

### Subjects

We observed the shapes and measurements of the foramen ovale using archival CT mastoid images. The sample size included 100 patients (50 males and 50 females) from KFHU radiological data system. Both left, and right foramina were examined, accounting for 200 foramina ovalia. We used a simple random selection of patients with mastoid CT. All patients with CT mastoid in KFHU and aged between 20-50 years were included in the study. Patients aged under 20 or more than 50 years, with neurological pathologies, or who underwent major neurosurgery were excluded from the study.

### Variables

In the study, various variables were examined to determine their impact on the foramen ovale (FO) in the human skull. The independent variable was sex, with female and male participants included in the study. The dependent variables consisted of several factors related to FO, including its shape, length, width, distance from the midline, and the presence or absence of a spur. Four different shapes of FO were considered, including almond, oval, round, and teardrop. Additionally, the length and width of both the right and left sides of FO were measured, as was the distance of FO from the midline. Finally, the presence or absence of a spur on FO was recorded. The controlled variables in the study included the participants' nationality, which was limited to Saudi individuals, and their age, which ranged from 20 to 50 years old.

### Data collection

Data were collected between January 2022 and February 2022. The measurements were made using a multi-detector row computed tomography scan (MDCT Scan, Somatom Definition, Siemens Healthcare, Forchheim, Germany). CT imaging parameters were 0.6mm section thickness, 120 KVp, and 150-180 mA. GE picture archiving and communication system (PACS) software was used to perform the measurements. The following/reported measurements of the brain were taken through axial images.

### Statistical analysis

The shape of the foramen ovale was distinguished as oval, round, almond, or teardrop. In addition, the presence of the bony spur was observed. The anteroposterior length and transverse width of the right and left FO and the distance from the midline (center) to the right and left FO was measured on axial images. The analyzed data are reported using the arithmetic mean (M), standard deviation (SD), standard error of the mean, and 95% coefficient interval (CI) and compared using the paired t-test. Paired t-test was used to compare the foramen dimensions of the left and right sides and morphometric variation between males and females. A p-value of <0.05 was considered statistically significant for all statistical analyses in this study. The data was analyzed for descriptive statistics and discriminant function analyses using the statistical package for the social science (SPSS) version 21 for windows (SPSS, Inc., Chicago, IL, USA).

## RESULTS

**Figure 1:**
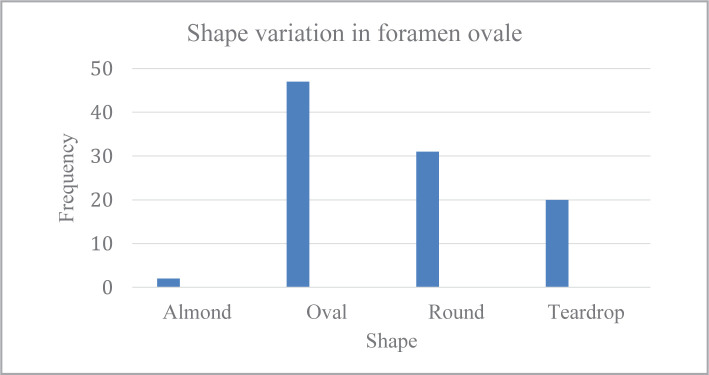
Shape variation in foramene ovale.

**Figure 2:**
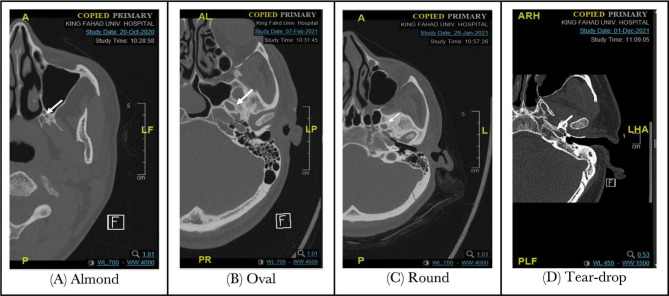
FO shape variations.

A total of 200 foramina ovalia from 100 patients were included in the analysis. They consisted of 50% male and 50% female, with a male-to-female ratio of 1:1. The shape of the foramen ovale was tested. The minority of patients had an almond shape of the foramen ovale (2%), followed by a teardrop shape (20%), a round shape (31%), and approximately half of the patients had an oval shape (47%). Table 1, Figures 1 and 2 A-D, present the shape variations in FO.

**Table 1:** Shape variations in the foramen ovale.

	Frequency	Percent	Valid Percent	Cumulative Percent
**Almond**	2	2.0	2.0	2.0
**Oval**	47	47.0	47.0	49.0
**Round**	31	31.0	31.0	80.0
**Teardrop**	20	20.0	20.0	100.0
**Total**	100	100.0	100.0	

**Figure 3:**
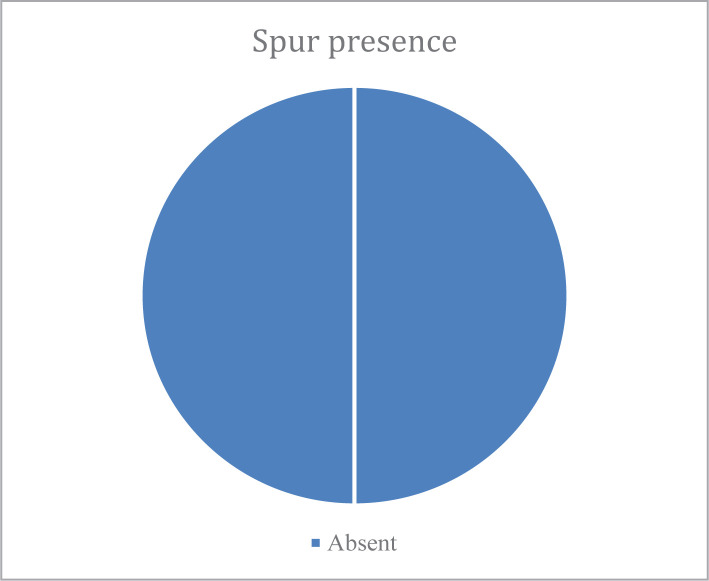
Spur presence.

Moreover, regarding the presence or absence of a spur in the foramen ovale, we noticed that this spur was not found in all examined patients. Table 2 and Figure 3 demonstrate the absence of a spur in the FO.

**Table 2:** Presence of spur in the foramen ovale.

	Frequency	Percent	Valid Percent	Cumulative Percent
**Absent**	100	100.0	100.0	100.0
**Total**	100	100.0	100.0	

The tested measurements of the foramen ovale included length, width, and distance from the midline bilaterally from both sides (right and left). The observed mean lengths were 6.462mm and 6.451mm on both the right and left sides, respectively. The mean width of the right side was 4.897mm, and 4.812mm of the left side. The reported distance from the midline was 2.4565mm on the right and 2.4290mm on the left. No statistically significant difference in the length, width, and distance from the midline between the right and left sides of FO was found. Table 3 shows the measurement of the foramen ovale on the right and left sides.

**Table 3:** Measurements of foramen ovale on the right and left side.

	Paired Differences					
	Mean	Std. Deviation	Std. Error Mean	95% Confidence Interval of the Difference		Sig.
				Lower	Upper	
**Length Rt**	6.462	1.6810	0.1681	6.1285	6.7955	0.889
**Length Lt**	6.451	1.6691	0.1669	6.1198	6.7822	
**Width Rt**	4.897	1.0631	0.1063	4.6861	5.1079	0.198
**Width Lt**	4.812	1.0848	0.1085	4.5967	5.0273	
**Distance from midline Rt**	2.4565	0.51275	0.05127	2.3548	2.5582	0.511
**Distance from midline Lt**	2.4290	0.60039	0.06004	2.3099	2.5481	

The dimensions of FO were compared between both sexes. The mean length of foramen ovale in males was 6.752mm and 6.161mm in females. The mean width was 5.008mm in males and 4.701mm in females. The mean distance from the midline was 2.530mm in males, while in females, it was 2.356mm. The differences in length, width, and distance from the midline between the two sexes were statically insignificant. Table 4 shows the measurements of the foramen ovale in males and females.

**Table 4:** Measurements of foramen ovale in males and females.

		Paired Differences					
		Mean	Std. Deviation	Std. Error Mean	95% Confidence Interval of the Difference		Sig.
					Lower	Upper	
**Length**	Male	6.752	1.7698	0.2503	6.249	7.255	0.069
	**Female**	6.161	1.4309	0.2024	5.754	6.568	
**Width**	Male	5.008	0.9915	0.1402	4.726	5.290	0.134
	**Female**	4.701	1.0405	0.1471	4.405	4.997	
**Distance from midline**	Male	2.530	0.5162	0.0730	2.383	2.677	0.092
	**Female**	2.356	0.5098	0.0721	2.211	2.500	

## DISCUSSION

The study analyzed CT mastoid images of 100 patients to observe and measure both left and right FO, accounting for 200 foramina ovalia. An equal number of males and females (50) were included to avoid bias. Furthermore, our study was conducted with a larger sample size than previous studies, which included 35 and 17 dried skulls [2,7]. Besides, involving data from live patients with known age and health status makes our study more applicable for the management of related pathologies guidance.

Regarding shape variation, Farooq [2], Yanagi [8], and Honnegowda [9] reported an oval-shaped FO observed in the majority of cases. Additionally, in our study, up to 47% of observed FO have an oval shape appearance. These shape variations are explained by many developmental reasons [7].

Moreover, as mentioned by Ray et al. [7], 74,3% of FO had oval shapes, while 25.7% varied because of developmental reasons, including a spine on the FO’s margin of 4.2%, tubercle protruding from a margin of 4.2%, a bridge-like spur 2.8% or a slit-like narrow shape 1.4%. Likewise, in the study by Farooq et al. [2], 5% had a bony spine, and 3% had a bony plate. In contrast, no spur was observed in this study.

The results of the present study indicate that differences between the right and left sides were insignificant in the length and width of FO, which is consistent with the result of many studies, including Liu et al.[7,9-15]. In our study, the mean length of FO was 6.46 mm ± 1.68 mm on the right side and 6.45 mm ± 1.66 mm on the left side, whereas these values in previous studies were 7.59mm ± 1.35mm and 7.68 ± 1.51, and 7.74 mm ±1.94 mm and 7.60 mm ±1.25 mm respectively [9,10]. Furthermore, the mean width on the right side was 4.89 mm ± 1.063 mm and on the left side 4.812 mm ± 1.084 mm, whereas Ray et al. [7] reported 3.21± 1.02 and 3.29 ± 0.85 in their study. In addition, the mean width was 3.4 mm and 3.8 mm on the right and left sides, respectively, according to a study conducted by fluoroscopically assisted laser targeting of FO in New York [16].

In the current study, we compared the dimensions of FO between males and females. The mean length was 6.752 mm in males and 6.161 mm in females, while the mean width was 5.008 mm in males and 4.701 mm in females. The mean distance from the midline was 2.530 mm in males and 2.356 mm in females. The differences in dimensions between the two sexes were found to be statistically insignificant (P-value >0.05), which is consistent with previous studies [7,8].

Future studies could potentially benefit from exploring additional variables to study multiple anatomical variations of FO, such as emphasizing the role of age. Moreover, including participants from different regions of Saudi Arabia would help to avoid a limited sample size and increase the generalizability of the findings to the entire Saudi population.

## CONCLUSION

Nearly half of the patients had an oval-shaped FO, and there was no documentation of spur presence in all examined patients. Finally, no statistically significant differences in the length, width, and distance from the midline were reported between right and left FO and both sexes.

This study can be considered a principal reference. These results can serve as a principal reference for future studies on FO morphological variation and its clinical applications.
